# 51 Neurological involvements in childhood-onset systemic lupus erythematosus: clinical, therapeutic and evolutionary profile

**DOI:** 10.1093/rheumatology/keac496.047

**Published:** 2022-10-07

**Authors:** Ourida Gacem, Zoulikha Zeroual, Zakia Arrada, Moussa Achir, Mohamed Samir Ladj

**Affiliations:** 1 Pediatrics Department Hospital Djillali Belkhenchir Birtraria Algiers Algeria; 2 Pediatrics Department of University Hospital Center Nefissa Hamoud Hussein Dey Algiers Algeria

**Keywords:** Neuropsychiatric, Systémic Lupus Erythematosus, Pediatrics, prognosis

## Abstract

**Backgrounds:**

Pediatric systemic lupus erythematosus (pSLE) is an autoimmune disorder, characterized by more severe multi-system involvement than in adults. The neurological and psychiatric manifestations of SLE are a heterogeneous set of clinical manifestations grouped under the term “Neurolupus”.

**Objective:**

We aimed to assess neuropsychiatric manifestations in childhood-onset Systemic Lupus Erythematosus (cSLE) in Algeria and to determine the clinical, therapeutic, evolutionary profiles and their correlations with the activity disease and the prognosis.

**Methods:**

**This was** a prospective, multicentre and descriptive study for 36 months (January 2015 - December 2018) at the department of Pediatrics of University Hospital Nefissa Hamoud ex Parnet Algiers. Children less than16 years of age fulfilling the American College of Rheumatology SLE criteria were included. Disease activity estimated by Systemic Lupus Erythematosus Disease Activity index (SLEDAI) whose use has been validated in children and damage index based on Systemic Lupus International Collaborating Clinics (SLICC) score were determined.

**Results:**

A total number of 83 patients, who had been diagnosed with cSLE, including 14 males (16, 9%) and 69 females (83.1%), were enrolled in the present study. Out of these 83 patients, 16 (19.3%) cases were younger than 6 years, 18 (21.7%) were 6–10 years, and 49(59%) were older than 10 years. The mean ages at lupus onset and diagnosis were 10, 12 ± 3, 88 and 11, 3 ± 3, 62 years respectively. Thirty-four patients (41%) developed neurological disease during this study. Neurological damage was polymorphic: psychosis and headache (38.2%), seizure (35.2%), motor deficit (32.4%), Other neurological manifestations included posterior reversible encephalopathy syndrome (PRES), Broca's aphasia, Guillain Barré Syndrome, Charles Bell paralysis (32.4%).

Neurological involvement was significantly correlated with the presence of anti-phospholipid antibodies (APL) (*p* = 0.00002), severe forms (*p* = 0.0006) and SLEDAI disease activity (*p* = 0.0001). Our patients received boluses of corticosteroids at the start and then cyclophosphamide.

The outcome was fatal for seven patients knowing that the overall number of deaths in our series was 9 deaths with a highly significant correlation according to the bivariate analysis between neurolupus and the death variable (*p* = 0.02).

Our study demonstrated that neurolupus in children was a predictive index of severity and prognosis based on multivariate analysis with an odds-ratio of [40.965 (1.470–1141.709)].

**Conclusion:**

Neuropsychiatric involvement complicates SLE in > 50% of cases. Pediatric neurolupus would be very heterogeneous and severe according to the few published pediatric series. It has a high lupus activity. It can be considered as a predictive factor of severity and prognosis. Neurolupus should be investigated at each lupus flare-up for early management in order to prevent any irreversible complication.

